# Age-Related Renal Microvascular Changes: Evaluation by Three-Dimensional Digital Imaging of the Human Renal Microcirculation Using Virtual Microscopy

**DOI:** 10.3390/ijms17111831

**Published:** 2016-11-02

**Authors:** Noriko Uesugi, Yoshihito Shimazu, Kazunori Kikuchi, Michio Nagata

**Affiliations:** 1 Department of Kidney and Vascular Pathology, Faculty of Medicine, University of Tsukuba, Tsukuba 305-8571, Japan; nagatam@md.tsukuba.ac.jp; 2 Laboratory of Food and Physiological Sciences, Azabu University, Sagamihara 252-5201, Japan; shimazu@azabu-u.ac.jp; 3 Division of Pathology, Tsukuba Medical Center, Tsukuba 305-0005, Japan; k-kikuchi@tmch.or.jp

**Keywords:** human renal microcirculation, healthy aging, paraffin-embedded sections, three-dimensional imaging, virtual microscopy

## Abstract

The renal microvasculature is targeted during aging, sometimes producing chronic kidney disease (CKD). Overdiagnosis of CKD in older persons is concerning. To prevent it, a new concept of “healthy aging” is arising from a healthy renal donor study. We investigated the renal microcirculatory changes of three older persons and compared them with that of one patient with nephrosclerosis using a three-dimensional (3D) reconstruction technique that we previously developed. This method uses a virtual slide system and paraffin-embedded serial sections of surgical material that was double-immunostained by anti-CD34 and anti-α smooth muscle actin (SMA) antibodies for detecting endothelial cells and medial smooth muscle cells, respectively. In all cases, the 3D images proved that arteriosclerotic changes in large proximal interlobular arteries did not directly induce distal arterial change or glomerulosclerosis. The nephrosclerotic patient showed severe hyalinosis with luminal narrowing of small arteries directly inducing glomerulosclerosis. We also visualized an atubular glomerulus and intraglomerular dilatation of an afferent arteriole during healthy aging on the 3D image and showed that microcirculatory changes were responsible for them. Thus, we successfully visualized healthy aged kidneys on 3D images and confirmed the underlying pathology. This method has the ability to investigate renal microcirculatory damage during healthy aging.

## 1. Introduction

The microcirculation of the kidney is essential for controlling glomerular filtration and tubular reabsorption and for regulating the medullary concentration gradient [1]. Dysfunction of the renal microcirculation directly induces glomerular, tubular, and interstitial damage. Benign nephrosclerosis, or arterio-/arteriolonephrosclerosis, a disease of renal microcirculation, develops in response to hemodynamic changes associated with chronic hypertension, aging, obesity, diabetes, and chronic kidney disease (CKD) [1]. Characteristic changes are hyaline degeneration of arterioles, intimal fibrous thickening with medial atrophy of the small arteries, segmental and global sclerosis of glomeruli, and interstitial fibrosis [2]. These changes are also typical morphological changes associated with aging [3]. In other words, aging is one of the most popular causes of nephrosclerosis.

Aging is an inevitable biological process that results in structural and functional changes. The kidney is one of the most susceptible organs to aging. One-half of adults over the age of 70 years have a low estimated glomerular filtration rate and are diagnosed as having CKD [4]. A biopsy study of transplant donors’ kidneys [5] proved that nephrosclerosis is observed in >50% of kidneys of persons >60 years old.

In this study, however, many older donors were systematically evaluated as “healthy” or at least “healthy for age” before biopsy. It is concerning that too many older patients could be unnecessarily labeled as “diseased” and treated without any proven clinical benefit [6]. Elderly persons with early-stage CKD might better be evaluated as undergoing “normal aging” or “healthy aging”. To prevent over-diagnosis, it is necessary to distinguish “healthy aging-related morphological changes” or “less harmful changes” from “harmful changes that cause advanced renal dysfunction”. To confirm that this is a good idea, we must first evaluate the direct relation between the renal microvasculature and its relevant tissue damage. Our “renal microvascular three-dimensional (3D) imaging” technique [7] might be a good tool for determining the difference.

In a previous article [7], we proposed 3D reconstruction of the human renal microvasculature using paraffin-embedded surgically removed tissue and a vertical slide system. The advantage of this method is that it enables us to examine the direct connection between arteries and peripheral tissue and then identify the underlying pathology [7,8,9,10,11]. The purpose of this study was to investigate whether our 3D imaging could distinguish less harmful renal vascular changes from the dangerous changes that induce renal dysfunction using 2D and 3D images of human tissue.

## 2. Results

### 2.1. Clinical and Pathological Characteristics of Four Cases

The clinical features and pathological characteristics of four patients (all older Japanese men) are shown in Table 1. In three of them, we did not detect renal disease, proteinuria, hypertension, diabetes, or hyperlipidemia. Patient 4 (case 4) was diagnosed as having hypertensive nephrosclerosis with mild renal dysfunction and hyperlipidemia. Among the four patients, he had the highest percentages of glomerulosclerosis and interstitial fibrosis. Patient 3 (case 3) had larger glomeruli than patient 1 (case 1). All patients except patient 4 (case 4) had clear cell carcinoma. Patient 4 had chromophobe carcinoma. All tumors were in stage I with no metastasis and no multifocal lesions.

### 2.2. Original 2D Images

**Figure 1:**
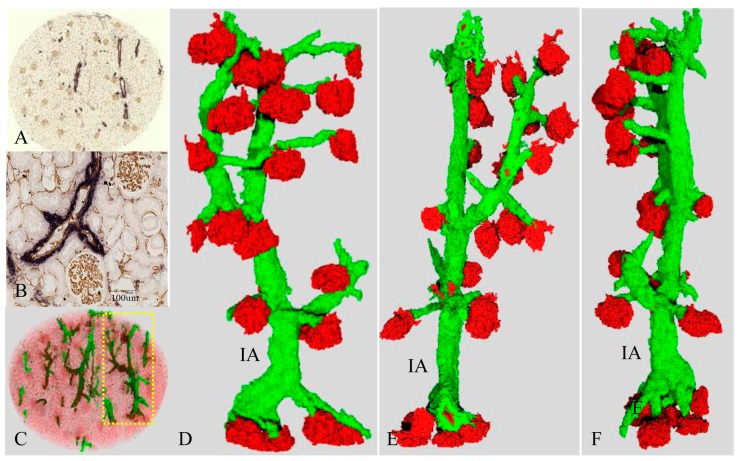
Vascular tree with glomeruli and the corresponding two-dimensional (2D) views in the patient with minimal Vascular Damage (Case 1). (**A**,**B**) original slice of a circular 3 × 3 mm core specimen with double immunostaining using anti-CD34 (**brown**) and then anti-SMA (**blue**); (**A**) and its magnified image (**B**); (**C**) three-dimensional (3D) reconstruction of the vascular tree; (**D**–**F**) an extracted vascular tree of yellow dotted circle of (**C**). IA is an interlobular artery; (**E**,**F**) rotated images of (**D**); and (**D**–**F**) original brown and blue correspond to **red** and **green** in the red-green-blue 3D images. Glomeruli can be identified as red figures. Arteries and arterioles are extracted as tubular formations of green SMA-positive smooth muscle cells. Interlobular arteries bifurcate and give rise to afferent arterioles, some of which branch off directly from large interlobular arteries (**D**–**F**).

In the original 2D images, glomeruli were seen as tufts of capillaries positive for CD34 (brown) (Figure 1A,B). Arteries were identified as lumens with endothelial cells positive for CD34 (brown) surrounded by strongly expressed α-smooth muscle actin (SMA) (blue) in the media (Figure 1A,B).

### 2.3. Three-Dimensional Reconstruction of the Vascular Tree in the Presence of Minimal Vascular Damage (Case 1)

Figure 1C is a reconstructed 3D image of the vascular tree, which is a continuous arterial structure composed of interlobular arteries and afferent arterioles in case 1. Figure 1D–F are 3D images of the isolated single vascular tree with accompanying glomeruli (corresponding yellow dot circle in Figure 1C. In the 3D image, red corresponds to the original brown of the CD34-positive endothelial cells, and green corresponds to the original blue of SMA-positive smooth muscle cells. Arteries are identified as green tubes. Glomeruli are expressed as red ovals. The vascular tree can be freely rotated, thereby enabling assessment of the running pattern, the length and branching angles of arteries, and the shape and size of glomeruli.

In case 1, the interlobular arteries showed no distortion and a regular distribution of green smooth muscle cells as seen by inspection (Figure 1C–F). The corresponding 2D images showed minimal intimal thickening (intima/media ratio: range, average; 0.03–0.3, 0.2 ± 0.12), preserved inner lumens (diameter: range, average; 9–41 μm, 20 ± 8 μm), a, and no atrophy of the media (Figure 1A,B). The 3D rotated images proved that most arterioles ran straight and branched off from their interlobular arteries at nearly a right angle. In addition, their diameters were almost the same as seen by inspection. The diameter of the interlobular artery was rather preserved until its last branching. Most of the glomeruli revealed similar shapes and sizes.

### 2.4. Three-Dimensional Reconstruction of the Vascular Tree in the Presence of Mildly Damaged Arteries (Case 2)

**Figure 2:**
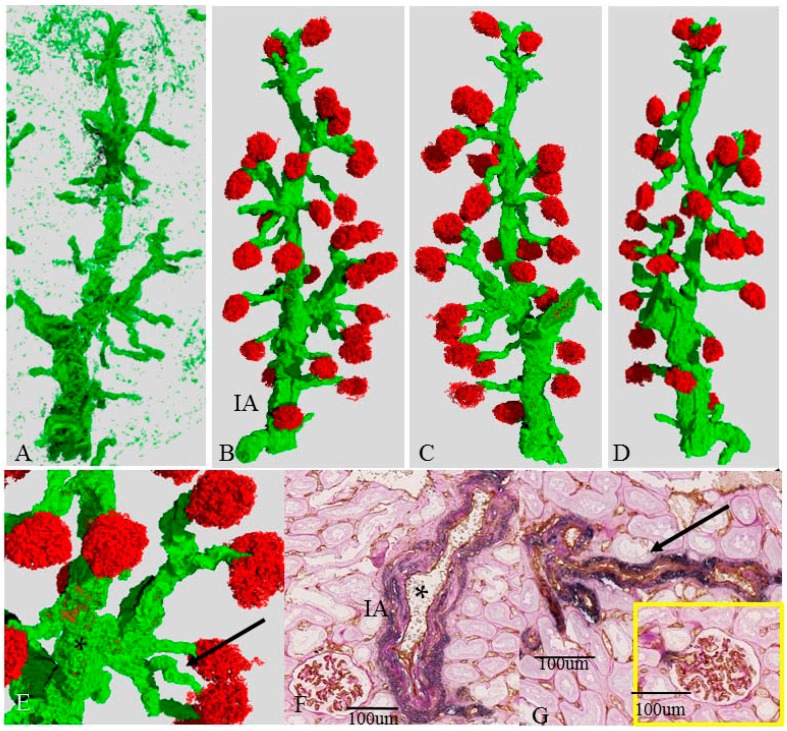
Vascular tree with mild change (Case 2). (**A**–**E**) single vascular tree with (**B**–**E**) or without (**A**) associated glomeruli; (**C**,**D**) rotated images of (**B**); (**E**) magnified view of (**B**). Red and green color correspond to CD34 and SMA in the 2D image, respectively. IA is an interlobular artery (**F**,**G**) corresponding 2D image that is double-immunostained with anti-CD34 (brown) and anti-SMA (blue) and then periodic acid-Schiff (PAS) stain. The asterisk (*) (**F**) and arrow (**G**) correspond to the same symbols in (**E**); (**A**–**E**) the interlobular arteries show irregular distribution of SMA and are mildly distorted; (**F**) corresponding 2D view shows that the media (**blue**) is atrophic, and SMA is irregularly distributed; the afferent arterioles are mildly distorted in the 3D view (**A**–**E**); whereas the corresponding 2D view (**G**) reveals regular media and segmental hyalinosis. The **yellow** circle represents a glomerulus supplied by this afferent arteriole.

Compared with case 1, the interlobular artery of case 2 showed irregular distribution of the smooth muscle cells and was mildly distorted in the 3D image as seen by inspection (Figure 2A–E).

The diameter of the proximal interlobular artery (102 μm) was larger than that of case 1 (92 μm), and the distal interlobular artery was much smaller than the proximal one. In the corresponding 2D image (Figure 2G), the interlobular arteries had fibrous intimal thickening (intima/media ratio: 0.4–1.5, 0.96 ± 0.0.82) and irregular medial atrophy, but a preserved lumen. Many afferent arterioles in case 2 were mildly distorted in the 3D image as seen by inspection. (Figure 2A–E). The corresponding 2D image (Figure 2H) showed hyalinosis and mild luminal narrowing (Figure 2G). Many glomeruli had preserved morphology and were of similar size. There were eight atrophic or sclerotic glomeruli in this vascular tree. Six of them were located at the distal end of the vascular tree near the cortical surface.

### 2.5. Three-Dimensional Reconstruction of the Vascular Tree in an Older Patient (Case 3)

**Figure 3:**
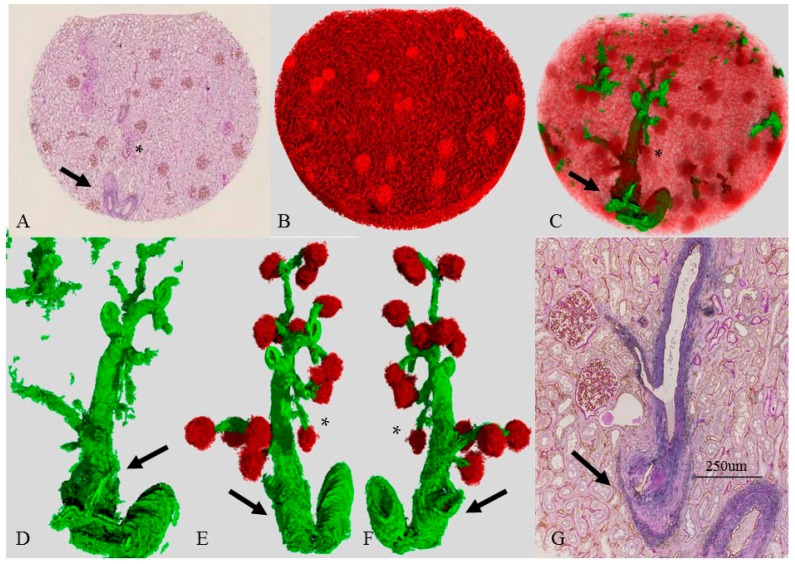
Vascular tree with decreased glomeruli (case 3). (**A**) Original 2D image of the section double-immunostained with anti-CD34 (**brown**) and anti-SMA (**blue**) and then PAS stain; (**B**) 3D reconstructed image of CD34. Glomeruli are round. Capillaries resemble a mesh network (**red**). Density of glomeruli is low; (**C**) 3D image of the vascular tree; (**D**–**F**) A single vascular tree with (**E**,**F**) or without (**D**) associated glomeruli; **Red** and **green** represent CD34 and SMA, respectively. All of the arteries show a regular SMA distribution and smooth surface. Arteries run straight. The branching angles of the afferent arterioles are sharp; (**G**) corresponding 2D image double-stained for CD34 and SMA with PAS stain. The diameter of the proximal interlobular artery is wide; and (**A**,**G**) corresponding 2D view reveals marked intimal fibrosis and medial atrophy; luminal area is preserved (**A**); and the arrow in (**G**) points to a bifurcation site and shows irregular intimal hyperplasia. Although the lumen seems narrow, the consecutive specimen reveals a large lumen, as seen in (**A**). The arrow and asterisk (*) correspond to the same symbols (**A**,**C**–**G**) and represent the interlobular artery and the atubular glomerulus in Figure 5, respectively.

In case 3, the proximal interlobular artery showed marked intimal thickening (intima/media ratio 2.1) and medial atrophy but with preserved lumens in the 2D image (arrow, Figure 3A,G). The distal interlobular artery and the afferent arteriole, however, revealed minimal intimal thickening (intima/media ratio 0.04–0.57, 0.34 ± 0.35) and no hyalinosis (Figure 3D–G). The luminal spaces were sufficiently large (14–41 μm). The accompanying glomeruli showed no definitive changes. Among the four cases, case 3 had the lowest glomerular density, and the average diameter was larger than those of cases 1, 2, and 3 (*p* < 0.05) (Table 1). The corresponding 3D image of the vascular tree (Figure 3B–F) showed that arteries and arterioles ran straight, and almost all glomeruli had similar shape and size (diameter 171–233 μm), but one (marked with *) was small (140 μm) and had an irregular shape compared with the others. The distal end of the interlobular artery had a rather small lumen. The branching angles of some arterioles from the interlobular artery were sharp compared with those in cases 1 and 2 (Figure 3G).

### 2.6. Three-Dimensional Reconstruction of the Vascular Tree in the Presence of Nephrosclerosis (Case 4)

**Figure 4:**
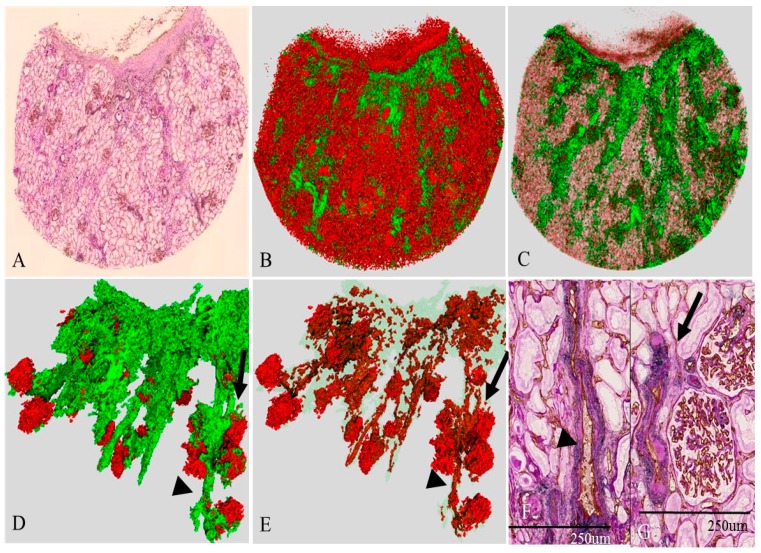
Vascular tree in Nephrosclerosis (Case 4). (**A**) Original 2D image of a section double-immunostained with anti-CD34 (**brown**) and the anti-SMA (**blue**) and then PAS stain; (**B**,**C**) 3D image of the renal microvasculature; (**D**,**E**) extracted view of the vascular tree; (**F**,**G**) 2D image. In the 2D image (**A**), differentiating the vessels from surrounding fibrotic interstitium is difficult. Thus, complete reconstruction of the vascular tree was not obtained, although some vessels can be identified; and the proximal interlobular artery (arrowhead, **D**–**F**) is smaller than that in case 2 (Figure 2) and case 3 (Figure 3). The smaller interlobular artery (arrow) (**G**) shows high hyalinosis and a reduced lumen.

Figure 4 shows the 2D and the 3D images of the renal microcirculation in the case of hypertensive nephrosclerosis with mild renal dysfunction. The 2D image (Figure 4A) highlights the prominent interstitial fibrosis and the presence of many sclerotic glomeruli near the capsule. In the corresponding 3D image (Figure 4B,C), SMA-positive areas were widely distributed in the cortex, especially near the renal surface and around each interlobular artery. Complete reconstruction of the vascular tree was difficult because the arterial media was not clearly distinguished from the surrounding interstitial fibrosis. In the 3D image, many interlobular arteries ran straight but were small and narrow (Figure 4D,E). The corresponding 2D view proved that the proximal interlobular artery (Figure 4F) showed no marked change in the media but mild hyalinosis in the intima. The distal interlobular arteries (arrows, Figure 4F,G) showed prominent hyalinosis and a decreased luminal area. The glomerular shape was irregular, and some glomeruli were enlarged. Glomerular density was decreased compared with those in cases 1 and 2 (Table 1).

### 2.7. Three-Dimensional Image of an Atubular Glomerulus (Case 3D)

**Figure 5:**
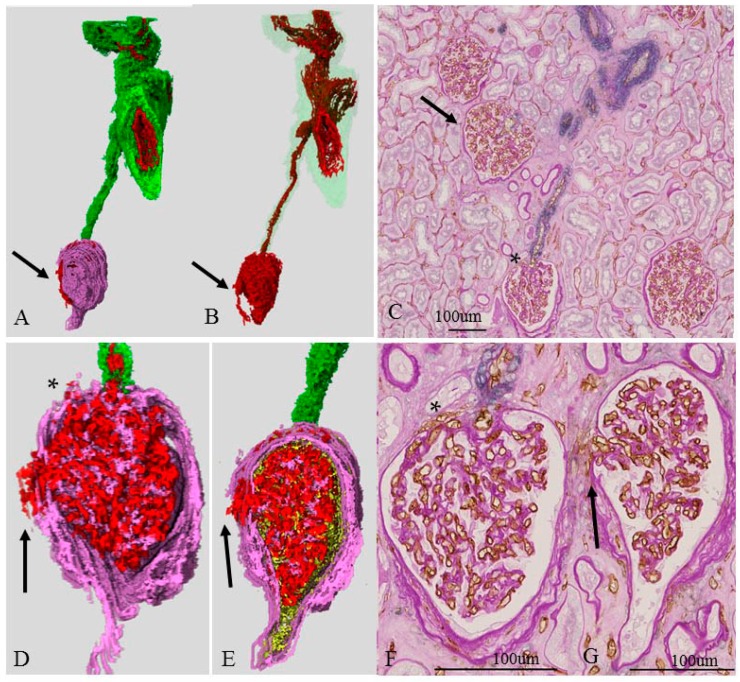
Three-and two- dimensional image of atubular glomerulus (Case 3). (**A**,**B**,**D**,**E**) 3D image of an atubular glomerulus with the interlobular artery and the afferent artery. **Red**, **green**, **pink**, and **yellow** represent CD34-positive endothelial cells, SMA-positive smooth muscle cells, PAS-positive basement membranes of the Bowman capsule and glomerulus, and epithelial cells of the glomerulus, Bowman’s capsule, and proximal tubule, respectively. These structures correspond to * in Figure 3A,C–F. (**C**,**F**,**G**) 2D image double-immunostained with anti-CD34 (**brown**) and SMA (**blue**) and then PAS stain. The interlobular artery has a large lumen (**C**). The afferent arteriole is long and has small lumen (**B**,**C**). The glomerulus (*) (**C**) corresponds to (**A**,**B**,**D**–**G**). The glomerulus (*) is smaller than the other glomerulus (arrow) (**C**) and shows shrinkage of the tuft (**F**,**G**); and the glomerulus has two exits (* and arrow) (**D**–**G**).

Figure 5 shows the 3D and 2D images of a single “atubular glomerulus” in case 3, which corresponds to the asterisks (*) in Figure 3E,F. Because of its long atrophic proximal tubule, the glomerulus was diagnosed as an “atubular glomerulus”. The 2D image (Figure 5C,F,G) proved that the glomerulus was small and exhibited shrinkage of the tuft and fibrous thickening of the Bowman capsule. The 3D and its corresponding 2D image confirmed the morphology of its supplying vessels. The interlobular artery had a large lumen with no definitive morphological changes in the 2D image (Figure 5C). In contrast, the afferent artery had a small diameter, and there was a great distance from the branching off of the interlobular artery (Figure 5A–C). The glomerulus had a small exit (*) near the afferent arteriole (Figure 5D,F), but we also found an extra exit apart from the afferent arteriole (arrows, Figure 5D–G). This extra exit is divided into several tiny capillaries soon after leaving the glomerulus and ran near the glomerulus (arrow, Figure 5A,B). Some were so tiny they could not be pursued (perhaps disappearing into the interstitium).

### 2.8. Three-Dimensional Image of a Single Glomerulus and Cystic Dilation of an Afferent Arteriole (Case 3) near the Atubular Glomerulus

**Figure 6:**
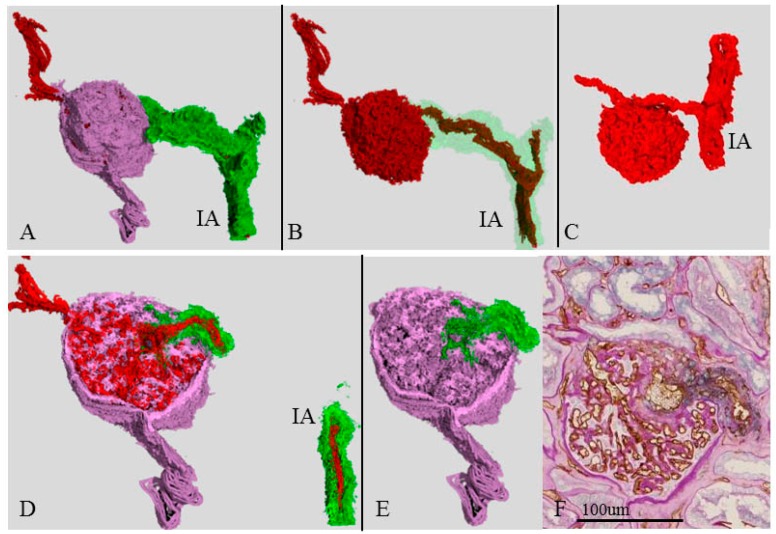
Three-dimensional image of abnormal efferent arteriole (Case 3). 3D images of a single glomerulus with abnormal efferent arterioles (**A**,**B**,**D**–**F**) in case 1; (**A**–**E**) **Red**, **green**, and **pink** are the CD34-positive endothelial cells, SMA-positive smooth muscle cells, and PAS-positive basement membranes of a Bowman capsule and glomerular basement membrane, respectively. IA is the interlobular artery. These structures correspond to the arrow in Figure 5C. The afferent arteriole is mildly distorted. Within the glomerulus, smooth muscle cells are still observed around the dilated afferent arteriole in the 3D view (**D**,**E**) and the 2D view (**F**); and the afferent arteriole is located apart from the efferent arteriole, whereas the arterioles were closely associated in case 1 (**C**).

Figure 6 shows the 2D and 3D images of a single glomerulus in case 3, which corresponds to the * in Figure 5G. The afferent arteriole was distorted and has an irregular lumen (Figure 6B) compared with case 1 (Figure 5C). Within the glomerulus, the afferent arteriole had surrounding abundant SMA-positive cells and was dilated. The exit of the efferent arteriole was apart from the entrance of the afferent arteriole in the 3D image (Figure 6A–E), whereas the 3D image in case 1 (Figure 6C) showed that the exits and entrances of the arterioles were closely located. The interlobular artery of case 3 had a small lumen compared with that in case 1 (Figure 6C).

## 3. Discussion

We examined 3D images of the renal microvasculature, especially glomeruli and their connecting vessels, in older persons with preserved renal function or mild renal dysfunction. We used the images to investigate the morphological changes seen during healthy aging.

The renal morphological changes in kidneys due to aging have been investigated using both 2D [12,13,14,15,16] and 3D [3] imaging. Age-related changes have been identified as mesangial matrix expansion, irregular foot process fusion, glomerulosclerosis [12,13,14,15], decreased number of glomeruli [12,13,14], glomerular hypertrophy [15], interstitial fibrosis [16], and arterial changes [3,17]. Arteries show hyalinosis and intimal thickening with medial atrophy [17]. Macroscopically, decreased kidney volume and size, reduced size of the cortex [18], formation of cysts, and cortical scars are also common findings in the aging kidney.

The abovementioned changes were found in each of our four cases in different degrees and morphology. Three had normal or nearly normal renal function for their age and had no complications. Compared with case 1, cases 2 and 3 showed more prominent sclerotic changes in the proximal interlobular arteries and increased sclerotic glomeruli and interstitial damage. Three-dimensional imaging, however, proved that the glomeruli and distal arteries and arterioles supplied by the damaged proximal interlobular artery had no severe morphological changes, although the increased number of sclerotic glomeruli had increased in the distal ends. Case 4, with severe intimal changes in the distal interlobular artery and many sclerotic glomeruli, exhibited only mildly impaired renal function. In the 3D images, the distal arteries and arterioles and glomeruli showed marked pathological changes. Our results revealed that the morphological changes in the large proximal interlobular artery or arcuate arteries did not directly induce severe renal damage, whereas small arterial changes were more critical to the development of glomerulosclerosis and interstitial damage. Nimomiya et al. [13] presented similar results based on Japanese autopsy cases with excellent statistical analyses. They showed that atherosclerotic changes in large arteries had no correlation with glomerular sclerosis and concluded that the arteries most susceptible to hypertension were the small ones (diameter < 150 μm). We successfully visualized this concept with 2D and 3D images.

We also visualized glomerular modifications in a case of healthy aging, in which an atubular glomerulus and cystic dilatation of the afferent arteriole were apparent. The glomerulus showed long, atrophic, proximal tubules—part of the definition of an atubular glomerulus [19]. Proximal tubules, known for their high rates of oxygen consumption and relative paucity of endogenous antioxidant defenses, are susceptible to many types of injury, including obstructive, ischemic, hypoxic, oxidative, and metabolic changes [19,20]. These injuries are believed to induce the formation of atubular glomeruli [19]. Our atubular glomerulus was considered to have an ischemic etiology because it showed tuft shrinkage and it was supplied by long, small afferent arterioles. Our 3D images also clearly identified an extra exit of this glomerulus. Inadequate glomerular circulation might have induced the formation of the extra exit and atrophic tubules. Our 3D image clarified the modifications of the glomerulus. The intraglomerular afferent arteriole was dilated and surrounded by abundant SMA-positive cells, and it was located apart from the efferent arteriole. These changes might also be induced by inadequate hyperfiltration because case 3 showed glomerular hypertrophy probably due to nephron loss. Modification of afferent and efferent arterioles has been proven in cases of aging. Such modifications include anastomoses and a direct channel between the afferent and efferent arterioles. We suspected that the glomerular modification might be a compensatory change in response to alteration of the glomerular circulation and possibly a morphological change due to aging.

Recent technical advances have enabled us to reconstruct high-resolution 3D images of the renal microvasculature, which have proved useful for investigating renal damage caused by an altered microvasculature [21,22,23,24,25]. Micro-computed tomography with contrast media can successfully quantify vessel density and vascular volume and has confirmed the rarefaction of renal capillaries in eNOS^−/−^ rat kidney [21]. Confocal microscopy with fluorescence micro-angiography proved the reduction of glomerular and peritubular capillary density [1] and the increase in capillary branching of glomeruli [22,23] in a diseased rat kidney. Recently, Torres et al. created 3D images of the whole kidney in cisplatin-treated mice and successfully reconstructed a complete nephron using multi-photon microscopy [24].

All 3D imaging of the renal microvasculature requires special tools. In addition, the techniques are rarely applied to humans and do not provide the underlying histology. Our technique is superior to those methods because it uses conventional paraffin-embedded sections and enables detection of the underlying histology. The disadvantages of our method are that it requires an expensive virtual slide system and human labor for reconstruction. In addition, the reconstructions are of limited size. Clearer figures of 3D reconstructions of the renal microcirculation may be obtained by X-ray micro-computed tomography [21], or scanning electron microscopy [25]. Our method, however, has great ability to reconstruct the boundaries between arterioles, glomeruli, and surrounding interstitium. It also enables comparisons between 3D and 2D histology, which is not achieved by any other 3D technique.

## 4. Materials and Methods

### 4.1. Tissue Preparation

Conventional surgical, formalin-fixed, paraffin-embedded tissue blocks from non-neoplastic renal parenchyma of four Japanese patients diagnosed with renal cell carcinoma were used in this study (Table 1). The tumor was diagnosed Kazumori Kikuchu, a pathologist who is one of our authors, and was classified according to Union for International Cancer Control (UICC) criteria. The institutional review board of Tsukuba Medical Center approved this data analysis (23 December 2011).

Conventional stains—e.g., hematoxylin-eosin, periodic acid Schiff (PAS), Masson trichrome, elastic-van Gieson—were used for morphological analysis and determination of suitable areas for tissue preparation. Details of tissue preparation are described elsewhere [7,8,9,10,11].

### 4.2. Immunohistochemistry and Microscopic Image Digitization

Double immunohistochemical staining with mouse anti-CD34 (Dako, Glostrup, Denmark) and anti-SMA (Dako) was performed to identify endothelial cells and smooth muscle cells of the arterial media (Figure 1A). The PAS stain was then applied to the sections to identify the tubular basement membranes, Bowman’s capsules, sclerotic glomeruli, and interstitial fibrosis (Figure 2F,G, Figure 3A,G, Figure 4F,G, Figure 5C,F,G and Figure 6G). Details of the tissue preparation are described elsewhere [7,8,9,10]. Each stained specimen was digitized using virtual slide microscopy (NanoZoomer RS™; Hamamatsu Photonics, Hamamatsu, Japan), using a 40× optical lens.

### 4.3. Image Registration, Segmentation of Tissue Components, Geometric Reconstruction

Image registration, segmentation of tissue components, and geometric reconstruction were performed using two software packages, Image-J (version 1.48, NIH, Bethesda, MA, USA) and TRI/3D-SRF (version 64, Ratoc System Engineering Co., Tokyo, Japan) as described previously [8,9,10]. Histological digitized images have a resolution of 0.92 µm per pixel, 5120 × 4096 pixels, 60 MB, and a 32-bit RGB TIFF format.

### 4.4. Conventional Histological Analysis of Surgical Specimens

Percentages of sclerotic glomeruli and tubular cortical interstitial fibrosis were assessed using image-processing software (WinRoof^®^; Mitani Corp., Tokyo, Japan) (Table 1). Using serial sections used for 3D reconstructions, the glomerular diameter was measured in 25–35 glomeruli. Diameter is defined as the largest one in the serial sections. Glomerular density was calculated as the glomerular number/mm^2^ cortex in a large surgical specimen. Intima/media ratio and luminal diameter were majorly in the serial 2D sections.

### 4.5. Statistical Analysis

Results are expressed as means ± standard error of the mean (SEM). Comparisons between two and among cases were performed using paired Student’s *t*-tests and one-way ANOVA, respectively. A value of *p* < 0.05 was considered to indicate statistical significance.

## 5. Conclusions

In conclusion, our 3D imaging of the renal microvasculature has the ability to prove a direct relation not only between the arteries and glomeruli but also between arteries and arterioles and between glomeruli and tubular structures. We successfully visualized 3D images of healthy aging microvascular changes. Our 3D imaging was of great help for clarifying the mechanism of microvascular renal injury in older persons and others with chronic renal disease.

## Figures and Tables

**Table 1:** Clinical characteristics of four cases.

Parameter	Case 1	Case 2	Case 3	Case 4
Age (years)	78	78	86	71
Height (cm)	171	164	160	168
Weight (kg)	63	53	54	68
BMI (kg/m^2^)	21	20	21	24
BUN (md/dL)	17	15	18	18
Creatinine (mg/dL)	0.91	0.96	0.80	1.27
eGFR (mL/min/1.73 m^2^)	61	59	69	43
CKD stage	2	3a	3a	3b
%Glomerular sclerosis	9	17	11	32
Creatinine at 6 month after surgery (mg/dL)	1.3	1.5	1.2	2.1
%Interstitial fibrosis	5	7	11	14
Mean Gl diameter (µm)	158 ± 18	161 ± 10	187 ± 22 *	162 ± 28
Gl density (/mm^2^ cortex)	4.4	3.7	2.5	2.7
Tumor pathology	Clear cell ca. 3.0 × 3.0 cm	Clear cell ca. 4.7 × 4.7 cm	Clear cell ca. 3.8 × 3.9 cm	Chromophobe ca. 2.6 × 2.4 cm
Stage of tumor (UICC)	Stage I	Stage I	Stage I	Stage I

* *p* < 0.05 vs. Case 1, 2, 4. Abbreviation: BUN: blood urea nitrogen; eGFR: estimated Glomerular Filtration Rate; CKD: chronic kidney disease; Gl: glomerular; Ca: Carcinoma; UICC: Union for International Cancer Control.

## Abbreviations

CKD: Chronic Kidney Disease
3D: Three-Dimensional
2D: Two-Dimensional
SMA: α-Smooth Muscle Actin
